# Evaluation of factors predicting diminished ovarian reserve before and after laparoscopic cystectomy for ovarian endometriomas: a prospective cohort study

**DOI:** 10.1186/s13048-016-0241-z

**Published:** 2016-06-21

**Authors:** Rie Ozaki, Jun Kumakiri, Andrea Tinelli, Grigoris F. Grimbizis, Mari Kitade, Satoru Takeda

**Affiliations:** Department of Obstetrics and Gynecology, Juntendo University Faculty of Medicine, 2-1-1, Hongo, Bunkyo-ku, Tokyo, 113-8421 Japan; Department of Obstetrics and Gynecology, Division of Experimental Endoscopic Surgery, Imaging, Technology, and Minimally Invasive Therapy, Vito Fazzi Hospital, Ospedale Vito Fazzi, 73100 Lecce, Italy; Department of Obstetrics and Gynecology, Aristotle University of Thessaloniki, Tsimiski, 51 Street, Thessaloniki, Greece

**Keywords:** Anti-Müllerian hormone, Bologna criteria, Cystectomy, Diminished ovarian reserve, Endometriosis, Laparoscopy, Poor ovarian responder

## Abstract

**Background:**

Ovarian endometriomas affect a substantial proportion of women of reproductive age who may have a potential risk of diminished ovarian reserve (DOR) after ovarian cystectomy. Here, we investigated the risk factors for pre-surgical DOR in patients with ovarian endometriomas and for DOR after laparoscopic ovarian cystectomy for endometriomas and evaluated the feasibility of the pre-surgical prediction of post-surgical DOR based on the Bologna criteria.

**Methods:**

A total of 143 patients with ovarian endometriomas who underwent laparoscopic cystectomy from January 2009 to May 2015 at our hospital were prospectively enrolled and evaluated. Serum anti-Müllerian hormone (AMH) concentrations were measured pre-surgically and at 3 and 6 months after surgery. In accordance with the Bologna criteria, the patients whose AMH concentrations were <1.1 ng/mL before surgery and 3 or 6 months after surgery were classified into pre- and post-surgical adverse DOR (aDOR) groups, respectively.

**Results:**

Thirty-one (21.7 %) of 143 patients were classified as pre-surgical aDOR. Patient age and serum follicle-stimulating hormone level were significantly positively correlated with pre-surgical aDOR [odds ratios (ORs), 1.26 and 1.16; *p* < 0.001 and *p* = 0.003, respectively]. Among the remaining 112 patients, 38 patients (33.9 %) had post-surgical aDOR 3 and 6 months after surgery. Bilateral cystectomy was positively correlated with post-surgical aDOR (at 3 months: OR, 4.7; *p* = 0.001; at 6 months: OR, 3.71; *p* = 0.006); conversely, pre-surgical serum AMH concentrations were negatively correlated with post-surgical aDOR (at 3 months: OR, 0.65; *p* = 0.005; at 6 months: OR, 0.43; *p* < 0.001). The optimal cut-off point of pre-surgical AMH concentrations for predicting aDOR at 3 and 6 months in the patients undergoing unilateral cystectomy was 2.1 ng/mL. In contrast, the optimal cut-off points at 3 and 6 months in the patients undergoing bilateral cystectomy were 3.0 and 3.5 ng/mL, respectively.

**Conclusions:**

Our data suggest that the pre-surgical serum AMH concentrations and bilateral cystectomy are significant factors for the risk of aDOR following surgery and that predicting post-surgical aDOR according to the Bologna criteria could be feasible using pre-operative measurements of serum AMH concentrations.

## Background

The current laparoscopic procedures for ovarian endometriomas, which affects 17 %–44 % of women with endometriosis [1], include ovarian cystectomy, ablation of the cyst wall and fenestration of cysts [2]. According to the European Society of Human Reproduction and Embryology (ESHRE) guidelines, ovarian cystectomy is preferred for the secondary prevention of endometriosis-associated symptoms and for increasing the spontaneous pregnancy rate [3]. However, both the guidelines and previous studies suggested a possible post-surgical reduction in the ovarian reserve, which indicates that follicles are inadvertently removed by the stripping of the cyst wall [4–7]. The serum concentration of anti-Müllerian hormone (AMH) is a highly sensitive marker of ovarian reserve, and, owing to its stability, it can be used to evaluate ovarian reserve after surgery to treat ovarian endometriomas [8, 9]. Serum AMH concentrations are known to significantly decrease after cystectomy for ovarian endometriomas.

The ESHRE working group established the Bologna criteria in 2011, which proposed a definition for the term poor ovarian responder (POR) to controlled ovarian stimulation for assisted reproductive technology (ART) [10]. A recent study reported that, based on the Bologna criteria, women diagnosed with POR after cystectomy for ovarian endometriomas had significantly lower clinical pregnancy and live birth rates after in vitro fertilisation (IVF) than women who were idiopathic PORs [11]. The study suggested that some patients with ovarian endometriomas have a potential risk of diminished ovarian reserve (DOR) and becoming a POR following ovarian cystectomy. However, the potential incidence and the risk factors have not yet been adequately investigated.

The aims of the present study were to investigate the factors associated with DOR and the potential risk of becoming a POR before and after laparoscopic cystectomy of ovarian endometriomas, and to evaluate the feasibility of pre-surgical prediction of post-surgical DOR according to the Bologna criteria.

## Methods

### Patients and recruitment

Patients who were admitted to our hospital from January 2009 to May 2015 for symptomatic ovarian endometrioma with a cyst >4 cm in diameter were enrolled in the present study. The disorder was diagnosed using transvaginal ultrasound and pelvic magnetic resonance imaging. The inclusion criteria were as follows: 1) non-pregnant patients; 2) age under 45 years with a regular menstrual cycle; 3) absence of any leiomyoma involving the cavity or intramural leiomyoma >3 cm in diameter; 4) absence of any other bleeding diseases; 5) absence of endocrine disorders, including thyroid dysfunction, hyperprolactinemia and Cushing’s syndrome; 6) no previous history of abdominal surgery; 7) absence of malignant ovarian diseases and 8) no previous history of hormonal treatment within 3 months before blood collection. The diameters of the ovarian cysts were measured in all the patients by ultrasound before surgery.

### Definitions of pre-and post-surgical adverse DOR

In accordance with the Bologna criteria, AMH concentrations already decreased to <1.1 ng/mL before surgery were defined as pre-surgical adverse DOR (aDOR) with a potential risk of the patient becoming a POR. AMH concentrations that were >1.1 ng/mL before surgery but decreased to <1.1 ng/mL at 3 or 6 months after laparoscopic cystectomy were defined as post-surgical aDOR. None of the patients underwent postoperative hormonal therapy until 6 months after surgery.

### Measurements

The diameters of the ovarian cysts were measured in all patients using ultrasound before surgery; the concentrations of serum AMH, luteinising hormone (LH), follicle-stimulating hormone (FSH), estradiol and cancer antigen 125 (CA125) were also measured. In patients with bilateral ovarian endometriomas, the total diameter was calculated as the sum of the diameters of both cysts. Serum AMH concentrations were also measured at 3 and 6 months after surgery. The sera, obtained from the blood samples by centrifugation (1,400 × *g*, 10 min) to separate the cellular contents and debris, were transferred into sterile polypropylene tubes and then cryopreserved at −80 °C until analysis. The serum AMH concentrations were measured using an enzyme immunoassay kit according to the manufacturer’s instructions (EIA AMH/MIS; Immunotech, Marseille, France). The intra- and inter-assay coefficients of variation for the AMH assay were below 12.3 and 14.2 %, respectively.

### Surgical procedures

All surgical procedures were performed by the same skilled surgeon (J.K.). The patient was placed in a 30° reverse Trendelenburg lithotomy position under general anaesthesia, and endotracheal intubation was then performed, with a pneumoperitoneum created using CO_2_ insufflation with an umbilical 11-mm trocar (VersaStep™; Covidien, Mansfield, MA, USA), approached via the closed method and maintained at a pressure of 10 mmHg. Under observation using a 10-mm rigid laparoscope (Endoeye™; Olympus, Tokyo, Japan) inserted through the umbilical trocar, three additional sites for trocar (VersaStep™; Covidien) insertion were made as follows: two 5-mm sites at 2 cm above the anterior superior iliac spine and a 12-mm site on the left side of the umbilicus on the left axillary line. A uterine manipulator (Ethicon, Somerville, NJ, USA) was inserted in the uterus of the patient, and the severity of pelvic endometriosis was assessed using a scoring system according to the revised American Society of Reproductive Medicine (Re-ASRM) classification immediately before initiating the laparoscopic procedures [12]. Furthermore, patients underwent adhesiolysis of the obliterated cul-de-sac when necessary. After the cyst was aspirated and drained, adhesions between the cyst wall and the pelvic wall or bowel were lysed, and an 18-gauge infusion needle was used to inject diluted vasopressin (Pitressin®; Goldshield Pharmaceuticals, Croydon, Surrey, UK; 0.1 U/mL: 10 U diluted with 100 mL saline) from the inner surface of the cyst wall into the subcapsular space depending on the diameter of the incised ovarian cyst. The incision was further extended to strip the cyst wall from the surrounding normal ovarian cortex as gently as possible using two atraumatic grasping forceps for traction and counter-traction after identifying the cleavage plane. After completion of the excision of the cyst capsule, 3–0 absorbable sutures (PolySorb®; Covidien) were used for approximation of the ovarian edges and bleeding control. Electrocoagulation with a monopolar (30-W) needle knife for haemostasis was minimally used before approximation. The total duration of the surgery was calculated as the sum of the amounts of elapsed time between the skin incision and the start of the pneumoperitoneum, the intra- and extracorporeal manipulations and the skin closure after finishing the pneumoperitoneum.

### Statistical analyses

Statistical analyses were performed using the computer software package IBM SPSS Statistics version 20 (IBM, Armonk, NY, USA). A Kolmogorov–Smirnov test was performed to analyse the normality of the respective parameters. Unpaired Student’s *t*-tests or the Mann–Whitney *U*-test was performed to compare consecutive variables, and chi-square tests or Fisher’s exact test was performed to compare categorical variables. The results are expressed as the mean ± standard deviation and 95 % confidence interval (CI) or median and range. Attribution analysis of the factors influencing pre- and post-surgical aDOR was followed by forward stepwise variable selection; logistic regression analysis was performed to minimise the effects of confounding factors. Receiver operating characteristic (ROC) curves were used to obtain the area under the curve (AUC) as well as the sensitivity and specificity for the predictive value of pre-operative AMH. The highest Youden index (sensitivity + specificity - 1) was considered as the optimal cut-off point for outcome prediction. A *p* value of <0.05 was considered statistically significant. Among the patients, the cumulative spontaneous pregnancy rate of infertile patients was analysed using the Kaplan–Meier method. Patients who were lost to follow-up or who underwent ART during the observation period until 6 months after surgery were defined as censored data. The cumulative pregnancy rates between the groups were compared using the log-rank test. We also calculated odds ratios (ORs).

## Results

**Fig. 1:**
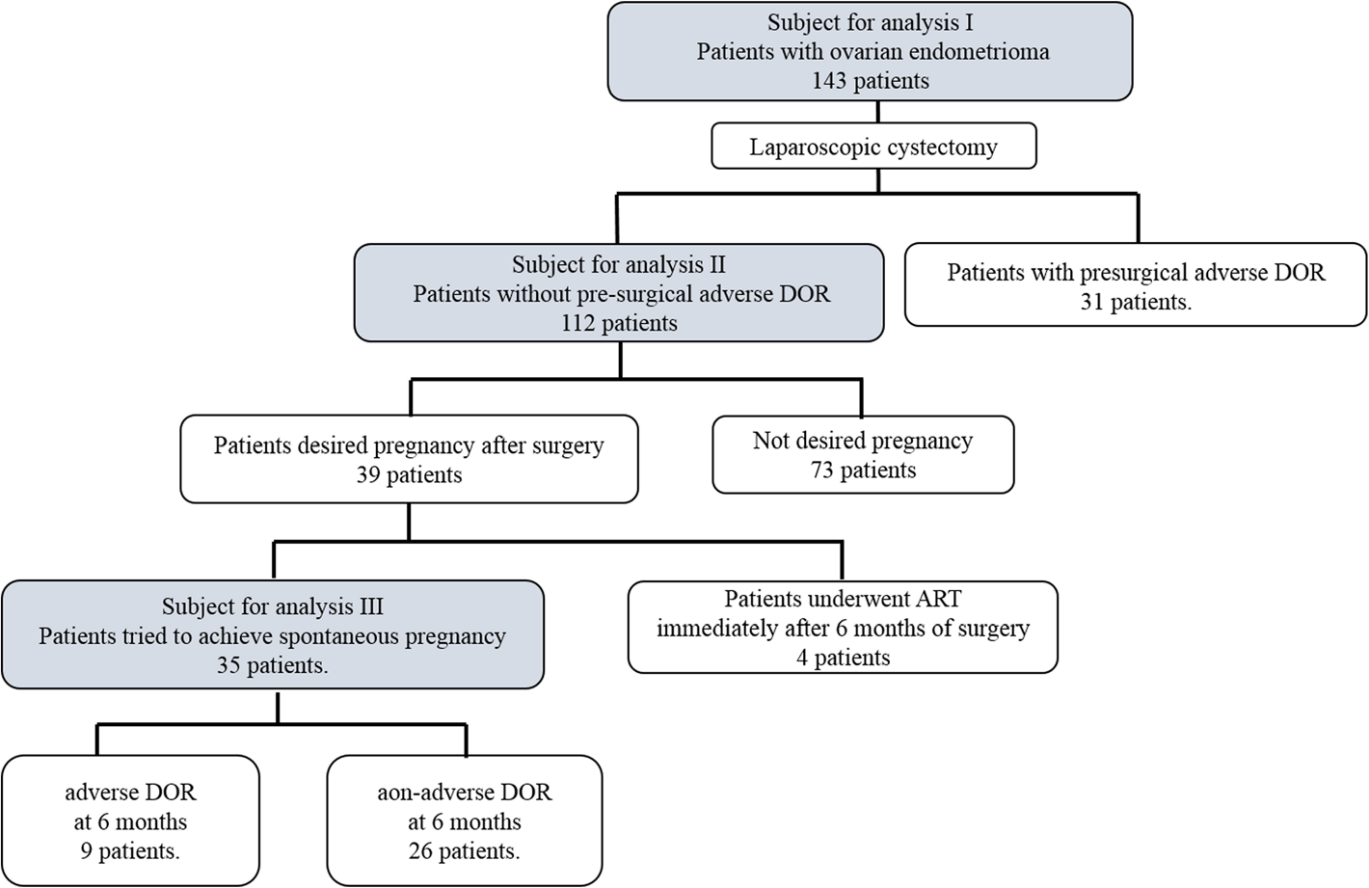
Diagram depicting flow of subjects through the study. Abbreviations: ART: assisted reproductive technology; DOR: diminished ovarian reserve

A flow diagram of the subjects analysed in this study is shown in Fig. 1. The following analyses were conducted: 1) analysis I, to evaluate influencing factors associated with pre-surgical aDOR among 143 patients who underwent laparoscopic cystectomy for endometriomas; 2) analysis II, to evaluate influencing factors associated with post-surgical aDOR at 3 and 6 months after surgery in 112 patients without pre-surgical aDOR and optimal cut-off points of the pre-surgical AMH concentration to predict post-surgical aDOR at 3 and 6 months after surgery by ROC curve analysis and 3) analysis III, to evaluate cumulative spontaneous pregnancy rates at 24 months after surgery in the aDOR and non-aDOR groups classified according to serum AMH concentrations at 6 months after surgery in 35 patents who tried to achieve spontaneous pregnancy after surgery.

### Assessments of pre-surgical aDOR in patients with ovarian endometriomas

Among the 143 patients, 31 (21.7) and 112 (78.3 %) were placed into the pre-surgical aDOR and non-DOR groups, respectively. Table 1 shows the pre-surgical patient backgrounds of both groups. Patient age, gravidity, parity, occurrence of bilateral cysts and serum LH, FSH and estradiol concentrations were significantly higher in the pre-surgical aDOR group than in the non-DOR group. Among these significant variables, age and serum FSH concentration were positively correlated with pre-surgical aDOR. The serum AMH concentrations of 31 patients in the pre-surgical aDOR group before surgery and at 3 and 6 months after surgery were significantly lower than those in the non-DOR group (Table 2).

**Table 1:** Factors associated with pre-surgical adverse DOR in patients scheduled for laparoscopic cystectomy for endometriomas

Characteristics	Univariate analysis			Multivariate analysis	
	Adverse DOR (*n* = 31)	Non-DOR (*n* = 112)	*P* value	OR (95%CI)	*P* value
Patient characteristics					
Age (years)	37.9 ± 4.1 (36.4–39.4)	33.5 ± 4.9 (32.6–34.4)	<0.001	1.26 (1.11–1.43)	<0.001
Gravidity (%)	0.5 ± 0.8 (0.2–0.7)	0.2 ± 0.5 (0.1–0.2)	0.02	−	−
Parity (%)	0.4 ± 0.6 (0.1–0.6)	0.1 ± 0.4 (0.03–0.2)	0.02	−	−
Body mass index (kg/m^2^)	22.0 ± 6.5 (19.6–24.4)	21.4 ± 5.0 (20.5–22.4)	0.64		
Laterality of cysts					
Unilateral (%)	10 (32.3)	64 (57.1)	0.01	−	−
Bilateral (%)	21 (67.7)	48 (42.9)			
Total size of ovarian cyst (mm)	83.1 ± 32.2 (71.3–94.9)	73.1 ± 27.5 (67.9–78.2)	0.09		
Pre-surgical serum hormone concentrations					
LH (mIU/mL)	9.7 ± 9.9 (6.1–13.4)	6.2 ± 8.4 (4.6–7.8)	0.047	−	−
FSH (mIU/mL)	10.3 ± 7.0 (7.7–12.8)	6.5 ± 3.5 (5.8–7.1)	<0.001	1.16 (1.05–1.28)	0.003
Estradiol (pg/mL)	134.5 ± 167.1 (73.2–195.8)	72.3 ± 87.2 (56.0–88.6)	0.01	−	−
CA125 (U/mL)	90.1 ± 104.7 (51.7–128.5)	68.9 ± 53.0 (58.9–78.8)	0.12		

Abbreviations: *CA125* cancer antigen 125, *CI* confidence interval, *DOR* diminished ovarian reserve, *FSH* follicle-stimulating hormone, *LH* luteinising hormone, *OR* odds ratio

Data are expressed as mean ± standard deviation (95 % CI) or number (percentage) of patients

**Table 2:** Comparison of pre- and post-surgical serum AMH concentrations between pre-surgical adverse DOR and non-DOR patients with ovarian endometriomas

	AMH (ng/ml)		*p* value
	Adverse DOR (*n* = 31)	Non-DOR (*n* = 112)	
Presurgical	0.7 ± 0.3 (0.6–0.8)	4.0 ± 2.8 (3.5–4.5)	<0.001
3 months after surgery	0.4 ± 0.4 (0.3–0.6)	2.3 ± 2.2 (1.9–2.8)	<0.001
6 months after surgery	0.5 ± 0.4 (0.4–0.7)	2.3 ± 2.1 (1.9–2.7)	<0.001

Abbreviations: *AMH* anti-Müllerian hormone, *CI* confidence interval, *DOR* diminished ovarian reserve. Data are expressed as mean ± standard deviation (95 % confidence interval) or number (percentage) of patients

### Factors influencing post-surgical aDOR after ovarian cystectomy

Postsurgical serum AMH concentrations at 3 months after surgery of all 112 patients in the pre-surgical non-aDOR group decreased below the pre-surgical concentrations. Among them, 38 (33.9) and 74 (66.1 %) were classified at 3 months after surgery into the post-surgical aDOR and non-aDOR groups, with serum AMH concentrations of 0.6 ± 0.4 and 3.2 ± 2.2 ng/mL, respectively. Table 3 shows the comparisons of the patient background characteristics and peri-surgical findings between the post-surgical aDOR and non-aDOR groups at 3 months after surgery. Univariate analysis revealed significant differences in age at surgery, the number of patients who underwent bilateral cystectomy, pre-surgical serum AMH concentrations and blood loss during surgery. Among these significant variables, bilateral cystectomy was positively correlated with aDOR at 3 months after surgery; conversely, the pre-surgical serum AMH concentration was negatively correlated with aDOR at 3 months after surgery.

**Table 3:** Factors associated with post-surgical adverse DOR at 3 months after laparoscopic cystectomy

	Univariate analysis			Multivariate analysis	
	Adverse DOR (*n* = 38)	Non-adverse DOR (*n* = 74)	*P* value	OR (95 % CI)	*P* value
Patient characteristics					
Age (years)	34.9 ± 5.0 (33.2–36.5)	32.8 ± 4.8 (31.7–33.9)	0.04	−	−
Gravidity (%)	0.2 ± 0.6 (0.01–0.4)	0.1 ± 0.9 (0.04–0.2)	0.67		
Parity (%)	0.2 ± 0.5 (0.2–0.3)	0.1 ± 0.3 (0.01–0.2)	0.47		
Body mass index (kg/m^2^)	21.8 ± 4.6 (20.2–23.3)	21.3 ± 5.2 (20.1–22.5)	0.67		
Laterality of cysts					
Unilateral (%)	13 (34.2)	51 (68.9)	<0.001	4.7 (1.93–11.5)	0.001
Bilateral (%)	25 (65.8)	23 (31.1)			
Total size of ovarian cyst (mm)	79.9 ± 35.0 (68.4–91.4)	69.6 ± 22.2 (64.4–74.7)	0.06		
Pre-surgical serum hormone concentrations					
LH (mIU/mL)	4.5 ± 4.0 (3.2–5.8)	7.0 ± 10.0 (4.7–9.3)	0.13		
FSH (mIU/mL)	6.4 ± 2.6 (5.6–7.3)	6.5 ± 3.9 (5.6–7.4)	0.96		
Estradiol (pg/mL)	79.4 ± 93.3 (48.7–110.1)	68.7 ± 84.3 (49.1–88.2)	0.54		
CA125 (U/mL)	65.5 ± 44.2 (50.9–80.0)	70.6 ± 57.1 (57.4–83.9)	0.63		
AMH (ng/mL)	2.8 ± 1.7 (2.2–3.4)	4.6 ± 3.1 (3.9–5.3)	0.001	0.65 (0.48–0.88)	0.005
Peri-surgical findings					
Re-ASRM score	60.9 ± 32.1 (50.4–71.4)	49.4 ± 27.5 (43.0–55.7)	0.05		
Total surgical duration (min)	91.5 ± 40.8 (78.1–104.9)	81.9 ± 32.0 (74.5–89.3)	0.18		
Total blood loss (mL)	40.2 ± 46.1 (25.0–55.3)	23.9 ± 26.8 (17.7–30.1)	0.02	−	−

Abbreviations: *AMH* anti-Müllerian hormone, *CA125* cancer antigen 125, *CI* confidence interval, *DOR* diminished ovarian reserve, *FSH* follicle-stimulating hormone, *LH* luteinising hormone, *OR* odds ratio, *Re*-*ASRM* revised American Society of Reproductive Medicine

Data are expressed as mean ± standard deviation (95 % CI) or number (percentage) of patients

At 6 months after surgery, serum AMH concentrations of 99 (88.4 %) of 112 patients of the pre-surgical non-DOR group decreased below the pre-surgical concentrations. Among the 38 patients in the aDOR group at 3 months after surgery, 30 (78.9) and 8 (21.1 %) were in the aDOR and non-aDOR groups, respectively, at 6 months after surgery. Among the 74 patients in the non-aDOR group at 3 months after surgery, 8 (10.8) and 66 (90.2 %) were in the aDOR and non-aDOR groups, respectively, at 6 months after surgery. Finally, at 6 months after surgery, 38 (33.9) and 74 patients (66.1 %) were in the aDOR and non-aDOR groups with serum AMH concentrations of 0.8 ± 0.7 and 3.0 ± 2.1 ng/mL, respectively. Table 4 shows comparisons of patient backgrounds characteristics and peri-surgical findings between the aDOR and non-aDOR groups at 6 months after surgery. Univariate analysis revealed significant differences in the number of patients who underwent bilateral cystectomy, pre-surgical serum AMH concentrations and Re-ASRM score. Logistic regression analysis revealed that bilateral cystectomy and the pre-surgical serum AMH concentrations significantly influenced the development of aDOR 6 months after surgery, which were the same significant factors as at 3 months after surgery.

**Table 4:** Factors associated with post-surgical adverse DOR at 6 months after laparoscopic cystectomy

Variables	Univariate analysis			Multivariate analysis	
	Adverse DOR (*n* = 38)	Non-adverse DOR (*n* = 74)	*P* value	OR (95 % CI)	*P* value
Patient characteristics					
Age (years)	34.6 ± 5.0 (33.0–36.3)	33.0 ± 4.8 (31.8–34.1)	0.09		
Gravidity	0.2 ± 0.6 (0.03–0.5)	0.1 ± 0.4 (0.03–0.2)	0.30		
Parity	0.2 ± 0.6 (0.1–0.6)	0.1 ± 0.3 (0–0.2)	0.15		
Body mass index (kg/m^2^)	21.7 ± 4.5 (20.2–23.2)	21.3 ± 5.2 (20.1–22.6)	0.73		
Laterality of cysts					
Unilateral	15 (39.5)	49 (66.2)	0.007	3.71 (1.45–9.51)	0.006
Bilateral	23 (60.5)	25 (33.8)			
Total size of ovarian cyst (mm)	75.4 ± 32.9 (64.6–86.2)	71.9 ± 24.4 (66.2–77.5)	0.52		
Pre-surgical serum hormone concentrations					
LH (mIU/mL)	4.7 ± 4.0 (3.4–6.0)	6.9 ± 10.0 (4.6–9.2)	0.18		
FSH (mIU/mL)	6.7 ± 2.4 (5.9–7.5)	6.4 ± 3.9 (5.4–7.3)	0.67		
Estradiol (pg/mL)	74.1 ± 89.3 (44.8–103.4)	71.4 ± 86.7 (51.3–91.5)	0.88		
CA125 (U/mL)	71.3 ± 48.5 (55.4–87.3)	67.6 ± 55.4 (54.8–80.4)	0.72		
AMH (ng/mL)	2.5 ± 1.2 (2.1–2.9)	4.8 ± 3.1 (4.0–5.5)	<0.001	0.43 (0.27–0.67)	<0.001
Peri-surgical findings					
Re-ASRM score	61.8 ± 33.7 (50.8–72.9)	48.9 ± 26.3 (42.8–55.0)	0.03	−	−
Total surgical duration (min)	89.8 ± 40.8 (76.4–103.2)	82.8 ± 32.3 (75.3–90.2)	0.32		
Total blood loss (mL)	37.4 ± 45.5 (22.5–52.4)	25.4 ± 28.0 (18.9–31.8)	0.09		

Abbreviations: *AMH* anti-Müllerian hormone, *CA125* cancer antigen 125, *CI* confidence interval, *DOR* diminished ovarian reserve, *FSH* follicle-stimulating hormone, *LH* luteinising hormone, *OR* odds ratio, *Re*-*ASRM* revised American Society of Reproductive Medicine

Data are expressed as mean ± standard deviation (95 % CI) or number (percentage) of patients

### Detection of optimal cut-off points of pre-surgical AMH concentrations

**Fig. 2:**
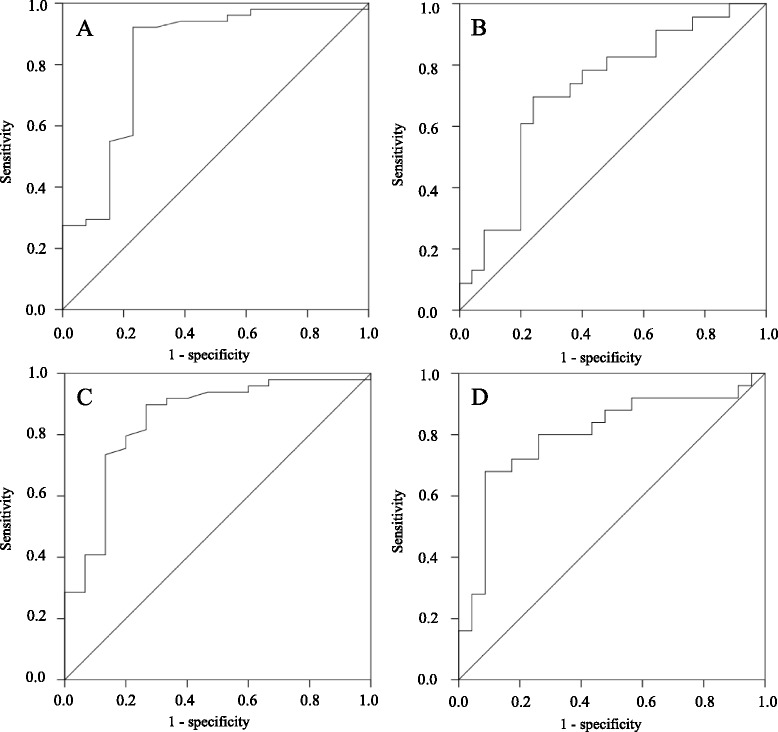
Receiver operating characteristic curves of pre-surgical anti-Müllerian hormone (AMH) concentrations for predicting diminished ovarian reserve at 3 and 6 months after surgery. The optimal cut-off points of the pre-surgical AMH concentrations at 3 and 6 months in the patients with unilateral cystectomy were 2.1 ng/mL (**a**, **b**). In contrast, the optimal cut-off points at 3 and 6 months in the patients undergoing bilateral cystectomy were 3.0 and 3.5 ng/mL, respectively (**c**, **d**)

Because the pre-surgical AMH concentrations significantly contributed to the likelihood of a patient having post-surgical aDOR at 3 and 6 months after surgery, as assessed by logistic regression analysis, we investigated the optimal cut-off points of the pre-surgical AMH concentration using ROC curves to predict post-surgical aDOR due to ovarian cystectomy. Figure 2 shows the ROC curves of the pre-surgical AMH concentrations of patients who underwent unilateral cystectomy and those who underwent bilateral cystectomy at 3 and 6 months after surgery. At 3 months after surgery, the optimal cut-off points of the pre-surgical AMH concentrations of the patients who underwent unilateral cystectomy and those who underwent bilateral cystectomy were 2.1 ng/mL [AUC, 0.83 (95 % CI, 0.68–0.97); sensitivity 92.2 %, specificity 76.9 %; *p* = 0.001; Fig. 2a] and 3.0 ng/mL [AUC, 0.72 (95 % CI, 0.57– 0.87); sensitivity 69.6 %, specificity 76.0 %; *p* = 0.01; Fig. 2b], respectively, and at 6 months after surgery, the respective optimal cut-off points were 2.1 ng/mL [AUC, 0.85 (95 % CI, 0.73–0.97); sensitivity 89.8 %, specificity 73.3 %; *p* < 0.001; Fig. 2c] and 3.5 ng/mL [AUC, 0.80 (95 % CI, 0.67–0.93); sensitivity 68.0 %, specificity 91.3 %; *p* < 0.001; Fig. 2d].

### Impact of post-surgical aDOR on spontaneous pregnancy after surgery

**Fig. 3:**
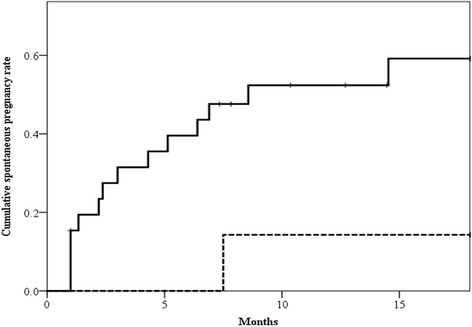
Cumulative probability of spontaneous pregnancy rate 6 months after laparoscopic cystectomy in the adverse diminished ovarian reserve (DOR) group (dotted line) and non-adverse DOR group (solid line)

Thirty-nine of the 112 patients in the pre-surgical non-DOR group desired to have a child following surgery. Four patients who desired to undergo ART immediately after the 6-month follow-up period were excluded from the analysis of the cumulative spontaneous pregnancy rate. Among the 35 patients remaining, 9 (25.7) and 26 (74.3 %) were placed in the post-surgical aDOR and non-aDOR groups, respectively, according to their serum AMH concentrations 6 months after surgery. Table 5 shows comparisons of patient background characteristics and serum AMH concentrations before surgery and at 3 and 6 months after surgery between the two groups. Although there were no significant differences in patient age and ovarian cyst laterality, the serum AMH concentration at each period was significantly lower in the aDOR group than in the non-aDOR group. Figure 3 shows the cumulative spontaneous pregnancy rates of the two groups at 18 months after the 6-month post-surgical measurements of serum AMH concentrations. The cumulative spontaneous pregnancy rate of the aDOR group was significantly lower than that of the non-aDOR group (14.3 % vs. 59.2 %, *p* = 0.04).

**Table 5:** Comparison of characteristics of adverse and non-adverse DOR infertile patients classified by serum AMH concentrations at 6 months after laparoscopic cystectomy

	Adverse DOR (*n* = 9)	Non- adverse DOR (*n* = 26)	*P* value
Age (years)	33 (29–39)	34 (26–39)	0.64
Laterality of cysts			
Unilateral (%)	5 (55.6)	16 (61.5)	0.76
Bilateral (%)	4 (44.4)	10 (38.5)	
Serum AMH concentrations (ng/mL)			
Before surgery	2.5 (1.7–3.4)	3.5 (1.5–10.5)	0.02
3 months after surgery	0.5 (0.1–2.3)	2.3 (0.4–6.1)	<0.001
6 months after surgery	0.6 (0.1–1.1)	2.1 (1.1–9.1)	<0.001

Abbreviations: *AMH* anti-Müllerian hormone, *CI* confidence interval, *DOR* diminished ovarian reserve

Data are expressed as median (range) or number (percentage) of patients

## Discussion

The results of the present study demonstrate that age and pre-surgical serum FSH concentrations were significantly associated with pre-surgical aDOR and that pre-surgical serum AMH concentrations and bilateral cystectomy were significant factors influencing the likelihood of a patient having post-surgical aDOR at 3 and 6 months after surgery. The optimal cut-off points of the pre-surgical AMH concentration to predict post-surgical aDOR due to ovarian cystectomy by ROC curve analysis were also determined. In addition, the cumulative spontaneous pregnancy rate was significantly lower in the post-surgical aDOR group than in the non-aDOR group at 6 months after surgery.

As evaluated by serum AMH concentrations, the reduction of ovarian reserve after ovarian cystectomy for endometriomas is inevitable. In our study population, 21.7 % of the patients were classified in the pre-surgical aDOR group based on the Bologna criteria. A decline in serum AMH concentration is highly correlated with the increasing age in women [13, 14]. Our data suggested that the ovarian reserve of aged patients with ovarian endometriomas was already diminished before surgery; thus, it is presumed that serum AMH concentrations in patients who have intrinsically low AMH concentrations would be further diminished. Therefore, the assessment of pre-surgical ovarian reserve for predicting further post-surgical decline of ovarian reserve could be more reliable if the serum AMH concentrations would be assessed in combination with a patient’s age and serum FSH concentration, which is a conventional ovarian reserve marker.

Several previous studies demonstrated that bilateral ovarian cystectomy is a significant factor associated with the decline of serum AMH concentrations after cystectomy for ovarian endometriomas [15–17]. The results of the present study revealed that pre-surgical AMH concentration is a crucial factor for the decline in serum AMH concentrations after unilateral and bilateral ovarian cystectomy. We believe that the post-surgical ovarian reserve after ovarian cystectomy for endometriomas depends on the pre-surgical potential of the ovarian reserve. In addition, it is thought that the optimal cut-off points of the pre-surgical serum AMH concentrations determined in the present study will be valuable for the pre-surgical prediction of DOR leading to patients becoming PORs. Previous studies have reported that the serum AMH concentrations in most patients who underwent a cystectomy for ovarian endometriomas decreased immediately after surgery and that the concentrations in some patients continuously decreased after the immediate reduction [18, 19]. The authors of these previous studies hypothesised that the continuous decline of the AMH concentrations could be attributed to vascular compromise, although the mechanisms are unknown. Our results showed that the cut-off point of the pre-surgical AMH concentrations in patients who underwent bilateral ovarian cystectomy at 6 months after surgery was increased compared with that at 3 months after surgery and that these cut-off points were higher at both 3 and 6 months compared with the concentrations of those who underwent unilateral ovarian cystectomy. We believe that these declines in the post-surgical serum AMH concentrations of patients who underwent bilateral cystectomy were due to a lower rate of recovery or a continuous decrease in AMH concentration because of the extended ovarian ischemia resulting from the bilateral surgery.

Meticulous surgical techniques for the excision of the capsule of ovarian endometrioma to find a correct cleavage plane and to achieve adequate haemostasis by coagulations using an energy devise are important for preserving the ovarian reserve [20]. In addition, an alternative management to cystectomy for ovarian endometriomas may be better for patients in whom further decline of the ovarian reserve should be prevented. Less reduction in serum AMH concentrations and antral follicle count (AFC) was observed when vaporising internal cyst walls using a CO_2_ laser as the source of energy compared with the excision of the cyst wall because the CO_2_ laser could vaporize only the filmy superficial internal lining up to 1.0 to 1.5 mm of the cyst wall [21]. However, higher recurrence rate after the vaporization remains debatable. Donnez et al. [22] reported that the ovarian volume and AFC did not significantly different between the operated ovary by combining excision and vaporization using CO2 laser and the contralateral unaffected ovary, with a low recurrence rate at short-term follow-up. In contrast, for patients who already have a decreased ovarian reserve before surgery and who desire pregnancy, ART may be more suitable because it has been reported that IVF outcomes, including oocyte retrieval and pregnancy, are promising even though the responsiveness of ovarian hyperstimulation may be decreased owing to the presence of the ovarian endometriomas [23]. Therefore, further research is required to examine whether these alternative managements instead of cystectomy are feasible for avoiding the impairment of future fertility in the predicted post-surgical DOR patients who have a potential risk for being post-surgical PORs.

The results of the present study demonstrated that the cumulative spontaneous pregnancy rate was significantly lower in the post-surgical aDOR group at 6 months after surgery than in the post-surgical non-aDOR group; the rate at 18 months after the 6-month post-surgical measurements of serum AMH concentrations was approximately 60 % in the post-surgical non-aDOR group. Although the Bologna criteria define PORs with respect to ART outcomes, our data indicate that post-surgical DOR based on the criteria are reflected in the spontaneous pregnancy rate after cystectomy of ovarian endometriomas; this rate might not have been as low if the patients had not become PORs post-surgically. Vercellini et al. reported that the spontaneous pregnancy rate after ovarian cystectomy for endometriomas in infertile patients ranged from 30 % to 67 %, and they concluded that the pregnancy rate 12 months after surgery in patients who underwent surgery for ovarian endometriomas could be hypothetically estimated to be not greater than 25 % owing to the multiple confounding factors of the studies in this field, including selection and publication bias [24]. Although the actual spontaneous pregnancy rate after ovarian cystectomy for endometriomas is debatable, the variability of this rate in previous studies might be related to the heterogeneity of the patients who experience difficulty getting pregnant owing to the degree of DOR in each study.

One limitation of our study is that we did not examine other markers of ovarian reserve. AFC is significantly correlated with serum AMH concentrations and is a reliable marker of the ovarian reserve [25]; however, the correlation was slightly altered when compared between operated and contralateral healthy ovary after cystectomy for unilateral ovarian endometrioma. Ercan et al. [23] demonstrated that AFC of the operated ovary significantly decreased compared with that of the non-operated ovary, although serum AMH concentrations did not significantly change at 3 months after laparoscopic unilateral cystectomy. Furthermore, meta-analysis for ovarian reserve after excisional surgery demonstrated that the ovarian reserve of the operated ovary, evaluated using AFC, was not significantly reduced after surgery, although AFC of the operated ovary after surgery was lower than that of the non-operated ovary [26]. Moreover, they stated that AFC is more accurate as a marker for ovarian reserve than AMH concentration because AFC directly indicates the ovarian reserve expressed by each single ovary. Conversely, AMH expresses the ovarian reserve of both ovaries, where a balancing effect of a healthy ovary may compensate for a reduced ovarian reserve in the contralateral affected ovary. Furthermore, the AMH values do not represent the quality of the oocytes. As the Bologna criteria define POR with respect to patient age, which is an oocyte quality-related factor, and to the responsiveness to ovarian stimulation, the outcomes of ART for these DOR patients may differ depending on the definitions used. Therefore, it is thought that other markers of ovarian reserve should also be evaluated to assess the reproducibility of our data.

## Conclusions

Laparoscopic cystectomy for ovarian endometrioma is an advanced surgical procedure that improves the symptoms of severe pain caused by conditions such as dysmenorrhoea and dyspareunia and results in a lower recurrence rate of ovarian cysts. However, a post-surgical reduction in ovarian reserve due to the excision of ovarian endometriomas is inevitable. Our study indicates that patients with pre-surgical aDOR could be identified and that the potential risk of being post-surgical PORs could be predicted by using optimal cut-off points of the pre-surgical AMH concentrations. These predictions will allow clinicians to select alternative therapies to prevent further decline of ovarian reserve, especially for infertile patients with ovarian endometriomas.

## Abbreviations

aDOR, adverse diminished ovarian reserve; AFC: antral follicle count; AMH: anti-Müllerian hormone; ART: assisted reproductive technology; AUC: area under the curve; CA125: cancer antigen 125; CI: confidence interval; DOR: diminished ovarian reserve; ESHRE: European Society of Human Reproduction and Embryology; FSH: follicle-stimulating hormone; IVF: in vitro fertilisation; LH: luteinising hormone; POR: poor ovarian responder; Re-ASRM: revised American Society of Reproductive Medicine; ROC: receiver operating characteristic.
